# Therapeutics and Materia Medica

**Published:** 1857-09

**Authors:** 


### THERAPEUTICS AND MATERIA MEDICA.

1. REPORT OF A CASE OF DEATH CAUSED BY SWALLOWING OIL OF TURPENTINE. By *John Maund, M.D.*—The following case seems, I think, worthy of recording, from my being unable to find any published account of death occurring from swallowing oil of turpentine. Christison, when speaking of this subject, says, "I am not aware that it has ever proved fatal." Taylor states, "I believe there is no case on record of destruction of life by turpentine." Other authors seem to agree in the same opinion, and neither Beck nor Guy, in their works on Medical Jurisprudence, treat of turpentine as a poison. From such testimony it evidently is manifest that death occurring from its administration must be very rare. Pereira and Christison both mention, on the authority of Professor Schubarth, "That two drachms of this oil administered to a dog produced immediate staggering, cries, tetanus, failure of the pulse and breathing, and death in three minutes."

Being anxious to ascertain if such effects were by any means constant, the following experiments were made by Dr. Youl and myself:

1st Experiment.—To a moderate-sized dog half an ounce of oil of turpentine was given; this produced immediately quickness of breathing, increased flow of saliva, frequent but ineffectual attempts to vomit, giddiness, and appearance of intoxication. The whole of these symptoms passed off in twenty minutes, and the dog appeared little worse for the dose.

2d.—To another dog was administered two ounces of oil of turpentine; similar symptoms to those before described immediately occurred; in addition, there was shaking of the limbs, and a greater amount of general prostration. Half an hour afterwards drowsiness became a prominent symptom, and the giddiness seemed to have increased, so that the dog could with difficulty stand. The eyes were much congested and everted, so that scarcely any portion of the cornea could be seen. The countenance presented the appearance of suffering and depression. The dog lay down on its side; frothy sputa kept running from the mouth, and the animal seemed to be dying; still, consciousness was never totally lost, for its attention could, though with difficulty, be attracted by speaking loudly, or by touching it. The following morning the dog seemed much better, and it gradually recovered from the effects of the turpentine.

3d.—To a third dog three ounces of oil of turpentine was administered; for the following quarter of an hour the only effects manifest were quickness of breathing, and increased flow of saliva, giddiness and general intoxicating effects then supervened; in half an hour the dog lay down on its side with its legs stretched out, and, excepting that the eyes were not everted, presented much the appearance of the second, and on the following day it gradually recovered.

4th.—To another dog four ounces of turpentine were then given; this, in two or three minutes, produced great excitement; respirations were 108 per minute; clear saliva kept streaming from both sides of the mouth, but no giddiness or sleepiness, as in the other cases, occurred. Half an hour after the turpentine was administered, the dog broke his cord and ran about, as if greatly excited, but he would come when called, and appeared perfectly tractable and docile.

From these experiments it would appear that the effects of oil of turpentine on dogs, described by Schubarth, like the following case of poisoning in the human subject, must be the exception rather than the rule.

The following account of a case of suicidal poisoning by oil of turpentine, I abridge from the depositions taken at the coroner's inquest, and from information that I obtained at the time of making the post-mortem examination.

E. H., a female of thirty years of age, and healthy appearance, had for some months been living with a man as housekeeper and mistress, but from his signifying his intention of leaving her, she became low-spirited, and for a few weeks before her death indulged freely in the use of stimulating drinks.

On the day of her death, a neighbor, with whom she was intimate, called to see her; she then appeared well, though a little excited, but by no means intoxicated. While conversing with the deceased, she noticed a soda water bottle, which she took up, as she thought it contained gin; at that time the bottle was nearly full, and contained turpentine. At the request of the deceased she went to fetch her some meat from the butcher's; on returning, shortly afterwards, not finding her in the kitchen nor answering to when called, she put down the meat and left the house. Four hours after this witness had left her, she was found dead, the meat and other things about the room being in the same order as the witness saw them when she returned with the meat.

The position of the body, which had not been moved when I saw it, about forty hours after death, at once suggested to my mind that death had occurred from strychnine, the results arising from which I am quite familiar with, having made four post-mortem examinations within the last three years, when death had taken place from this poison. The deceased evidently, immediately before death, was sitting on the side of the bed, and when found, seemed to have simply fallen backwards across the bed. The legs were rigid, and stretched out before the body, and the soles of the feet were concave; the arms were bent across the chest, and great force was required to straighten or remove them from this position, the biceps muscles being forcibly contracted and very hard. The body assumed the state of opisthotonos, and all portions of it were rigid, but the thighs least so. The eyes were open and prominent, and the pupils slightly dilated; the jaws were fixed, and could not be opened; the skin generally was pale, but of a livid hue in places, particularly about the head. The general appearance of the body was such as to give the idea that death had occurred suddenly from tonic spasm; there was no derangement of the dress or bedclothes to indicate convulsive action, and no external marks of violence. An empty pail was found on the floor close to the deceased, which probably she had placed there in case sickness should occur.

The internal organs of the body presented the usual appearance of a person dying from asphyxia. The membranes of the brain and upper portion of the spinal cord were found greatly distended, with very dark-colored blood of a sizy consistence, but it had no unusual smell. The brain was not softer than natural, but was, to a less extent than its membranes, congested with blood of the same character. The mucous membrane of the trachea was rendered quite arborescent by the ramification of a network of distended vessels, but in the interstices the membrane was of the usual color. The lungs were gorged with blood of the same dark appearance as was found in the membranes of the brain. The cavities on the right side of the heart were distended with blood, the left also contained a small quantity of blood; in both sides it was of equally dark color. The liver and kidneys were congested, but to a less extent than the organs before mentioned. The bladder was empty and firmly contracted, but appeared healthy. The stomach and bowels were the last part examined. Immediately on the stomach being opened, a most powerful smell of turpentine became evident, which had not before been recognized; this organ contained a small quantity of a thick fluid, which had the appearance of an emulsion made with turpentine and mucilage. The duodenum and upper portion of the jejunum were considerably congested, and the smell of turpentine was evident in all portions of the intestinal canal.

A further examination of the stomach and its contents showed that the mucous membrane was congested, and several very large vessels, injected with dark blood, were found passing from the cardiac to near the pyloric extremity, and in several places close to these vessels small ecchymosed patches existed. The contents of the stomach amounted to three ounces: it was of a semi-fluid character, and globules of what appeared to be oil of turpentine were seen to be intermixed with the more tenacious contents. Distilled water was then added to it, and thoroughly mixed together; three hours afterwards there was found swimming on the surface a limpid fluid, which was removed with a pipette, and was found to consist of oil of turpentine, of which there were six drachms. The remaining contents of the stomach were then examined for strychnine, but none was discovered in this, the parietes of the stomach, or mixed with the turpentine which had been removed. Under the microscope the solid contents were seen to consist chiefly of wheat and potato starch corpuscles.

The house was carefully searched as soon as the deceased was discovered, to ascertain if poison of any kind could be found, or if any vessel was soiled by any unusual fluid; neither, however, could be discovered. The bottle containing turpentine was found on the shelf, where the witness said she saw it before leaving to fetch the meat for deceased; the quantity removed from the bottle was about six ounces; whether the whole of this was swallowed, it is impossible to ascertain, but it seems highly probable that the portion taken was drank directly from the bottle. No smell of turpentine in the house, or suspicion of its having been taken, existed till the stomach was opened.

The deceased, it seems, had several times hinted that she would destroy herself, but this threat did not appear to have excited any serious apprehension that she intended carrying it into effect.—*Glasgow Med. Journal*, April, 1857.

ON THE EMPLOYMENT OF AMYLENE. By *M. Robert and others.*—M. Robert recently read at the *Académie de Médecine* an interesting report of a commission composed of MM. Malgaigne, Velpeau, and himself, upon a memoir on amylene submitted to the Académie by M. Débout. The report states that M. Débout's paper offers nothing new upon the subject, but confirms the observations already published by Snow, Giraldès, and Tourdes, establishing that amylene produces anæsthesia very speedily, without causing any painful sensation, or inducing that desire to cough or expectorate so often observed in the employment of chloroform. "During the entire procedure," says M. Débout, "the pulse remains large, full, and very rapid, the respiratory movements are deep, the skin warm, the face highly colored. In one word, there is an absence of those signs which indicate that the new agent can readily exert any effect upon the phenomena of organic life."

The reporter, in order to judge of this favorable account of amylene, made a number of trials with it himself, employing Charrière's apparatus, which, by covering both nose and mouth, prevents a loss of the vapor. One of the remarkable properties of amylene being its slight solubility in the blood, its vapor must be breathed in as concentrated a manner and as continuously as possible, under the risk of failing to produce insensibility, or of this being of too short duration. M. Robert believes that it is the want of observing this precaution, and from merely administering it on a sponge, that some surgeons have failed altogether, or have had to employ very large quantities of amylene. He has employed it in forty-four adults of both sexes. Most of the operations were but of short duration, such as opening abscesses or panaris, avulsion of nails, or amputation of toes; but some have been of a more important character, such as amputation of the thigh and arm, removal of the breast and the parotid gland, and extraction of a calculus from the prostatic portion of the urethra ; this last requiring a quarter of an hour. In none of these cases was any irritation of the mucous membranes produced. Most of them were rendered insensible in two or three minutes, a few not until six or seven ; while in three, after ten or twelve minutes' inhalation had been tried in vain, chloroform had to be resorted to. The agitation which so frequently precedes the action of chloroform was not observed. The countenance became more and more flushed. The eyelids remained widely open, and the fixed eyes were frequently carried upwards, beneath the upper lid. The head was thrown backwards, and sometimes the limbs became extended and stiffened. The pulse was very rapid, and, in one case, its intermittent and filiform character excited alarm. Respiration continued free, and the spasmodic closure of the jaws, with threatened suffocation, sometimes observed under chloroform, was never met with. It is an important fact, that amylene never gives rise to muscular resolution, and the insensibility it induces is of very short duration, unless the application be constantly repeated. After the operation, the restoration is rapid, the patient not continuing to suffer any uneasiness. Two young girls, however, exhibited a singular form of delirium, accompanied by sobbing and violent convulsive movements. One of them, subjected some time after to chloroform, exhibited the same symptoms. Thus, while amylene resembles ether and chloroform in its power of preventing pain, it differs from them in the rapidity and temporary duration of its action, and in not exerting any power on muscular contractility.

As to the question of danger, M. Robert observes that Dr. Snow's case has proved that, as in the use of chloroform, death may take place in consequence of a special predisposition of the economy of an unknown nature. The only question really is, whether there is less danger attaching to the employment of amylene. From various experiments he has performed upon animals, M. Débout concludes that there is; and he calculates that, if the dose of chloroform has to be doubled, to convert it from an anæsthetic into a toxical agent, that of ether has to be quadrupled, and that of amylene to be quintupled. The reporter, having repeated the experiments upon birds and rabbits, believes that this statement is accurate. Performing other experiments upon dogs, employing for that purpose a modification of Charrière's apparatus, he found that chloroform gave rise to the usual series of symptoms, from simple insensibility to complete muscular resolution, the animals always dying in the course of thirty or forty minutes. Under the use of amylene the same symptoms were observed as in man, but a relaxation of the muscles was never obtainable. Moreover, continuing the experiment, with the intention of killing the animals, these seemed to become habituated to its use, and recovered part of their sensibility. The inhalation was terminated at the end of an hour, and the animals soon began to walk, and were speedily restored. Having contrived an apparatus by which large quantities of amylene could be breathed in a concentrated form, the reporter at last succeeded in producing complete resolution of the limbs, and death followed in twenty minutes. While these experiments show that amylene is a much less powerful poisonous agent than chloroform, the reporter does not agree with M. Débout that it may, therefore, be more safely used in practice. It is, in fact, a leading point in the history of anæsthetics, that death has not supervened in man upon a successive and progressive evolution of the phenomena of intoxication, but that it occurs suddenly and unexpectedly, and in consequence of an unknown predisposition of the economy.

Amylene is therefore but another anæsthetic agent, to be placed side by side with ether and chloroform. Its action is prompt and of short duration, while its effects rapidly disappear. It is to be preferred in very short operations, when we wish simply to prevent or moderate the pain. Moreover, its not exciting irritation of the air-passages renders it a valuable agent when pulmonary lesions exist. It does not, too, give rise to vomiting or nausea, which are not infrequent after chloroform. This is important, especially in relation to children, enabling us to operate nearer their mealtime, and avoid the inconvenience of submitting them to long fasting, which they support very badly; nevertheless, vomiting does occur occasionally.

As the insensibility produced is of such short durability, and muscular contractility is only exceptionally influenced by it, amylene should not be employed for long and difficult operations, and especially those in which it is requisite to subdue the contractility of the muscles, as in dislocations, hernia, the diagnosis of abdominal tumors, &c. M. Tourdes has stated that muscular resolution may at last be obtained by a sufficient prolongation of the anæsthesia; but independently of the inconvenience that might result from the absorption of such large quantities of amylene, and the troublesome uncertainty the surgeon is kept in respecting the return to consciousness of his patient, it is by no means proved that such resolution would ensue upon a multiplication of the doses. Moreover, the reporter's experiments would seem to show that such repetition, providing the dose be not rendered excessive, tends to destroy the activity of the agent; while when resolution was obtained it was only shortly before death.—*Bull. de Thérap.*, May, p. 443.

M. Velpeau, as a member of the Committee, observed that he was no great partisan of amylene, and does not think, as far as his own experience goes, that it is likely to displace chloroform. Against its employment are its detestable smell, alike annoying to operator and patient, the little constancy of its effects, and the necessity of employing an apparatus. As to chloroform, he believes that the amount of danger attending its use has been grossly exaggerated. During the ten years he has employed it, in from five to six thousand cases, for operations at different ages and in both sexes, he has met with no accident whatever. No important accident has, indeed, occurred at any of the great Paris Hospitals. Moreover, how unjust, whenever a death occurs during or after an operation, to attribute it to chloroform! Employed with proper precautions, he does not regard it as a bit more dangerous than amylene, while it may be administered without any apparatus.

M. Giraldès, at a subsequent meeting of the Académie (*Gaz. Méd.* No. 21), gave a further account of his experience, having used amylene in the cases of seventy-nine children, varying from one to fourteen years of age, two drachms being in most cases the quantity employed. The time required for the production of the anæsthesia is usually about three minutes, but it varies much, both in different persons and in the same individual. No ill consequence has resulted, the anæsthesia taking place without reaction, or convulsions. In eight cases muscular rigidity was observed; and when this occurs the inhalations should be suspended awhile, and free respiration permitted. In six cases vomiting occurred. The anæsthesia lasted for a very short time, but it may continue for eight or ten minutes, and is easily kept up. Chloroform is a more powerful agent, but it is also a more dangerous one. It may throw feeble children into a state of cadaveric resolution; and the anæsthesia it induces is often so prolonged as to endanger life. On other occasions it induces spasmodic closure of the jaws, rigidity of the muscles of the neck and thorax, and all the symptoms of asphyxia, rendering artificial respiration necessary. It generally gives rise to vomiting, or other signs of irritation of the digestive organs. After the employment of amylene sensibility rapidly returns, and the children seem in nowise to suffer from it. It may be given soon after meals, and to enfeebled children, in whom chloroform would prove dangerous. It thus has, like chloroform, its special indications, and frequently presents indubitable advantages.

Dr. Kadlburger (*Win Wochenschrift*, No. 19) relates the results of his experience of the employment of amylene in seventy-two cases of tooth-extraction, he applying it by means of a sponge to both mouth and nostrils. He states that the amylene he employs emits a smell of over-ripe pears, and is nowise irritating to the respiratory organs. From one to one and a half minute's inhalation suffices for this purpose. The falling of the uplifted arm, as if wearied, is the signal for the discontinuance of the inhalation. No abnormal muscular movements or closure of the jaw were produced. The patient readily opened the mouth upon being requested to do so; and during the extraction most persons retained the consciousness of the application of the instruments. The pain, when felt at all, was very slight, and never at all approaching to the torture attendant upon ordinary tooth-drawing.—*Med. Times and Gazette*, June, 20, 1857.

